# Navigating the Impacts of Dementia: The Experience of Male Spousal Carers

**DOI:** 10.3390/healthcare11182492

**Published:** 2023-09-08

**Authors:** Edward Tolhurst, Bernhard Weicht

**Affiliations:** 1 School of Health, Science and Wellbeing, Staffordshire University, Stoke on Trent ST4 2DE, UK; 2 Department of Sociology, University of Innsbruck, 6020 Innsbruck, Austria; bernhard.weicht@uibk.ac.at

**Keywords:** dementia, care, male carers, masculinity, qualitative research

## Abstract

This article investigates the experience of male spousal carers for women living with dementia. While cultural discourses on care are highly gendered, social scientific research often addresses care relationships in gender-neutral terms. Setting out to address this matter, this qualitative research study incorporated semi-structured joint interviews with 10 couples in which a male spouse cared for a woman with dementia. The aim was to explore how couples negotiate relationships and care following a diagnosis of dementia. The focus of this paper is on the perspectives expressed in these joint interviews by the male carers. A thematic analysis was undertaken to establish the key content of the men’s accounts. Three principal themes were identified: making sense of the condition; treating dementia as a problem to be solved; and engaging with professionals and support. The gendered basis of experience for male carers is explored within these themes, demonstrating how societal norms of masculinity intersect with caring roles. The paper concludes that a nuanced research approach to dementia care must continue to be developed, accounting for how gender shapes personal responses to the navigation of care relationships. Practitioners and policymakers must also consider how gendered experience shapes the identities and strategies of male carers.

## 1. Introduction

People living with dementia and their carers encounter multiple emotional and practical challenges associated with a neurological disease that often affects memory, communication and behaviour [1]. While dementia and caring can present substantial difficulties to those experiencing the condition first-hand, they also present significant economic and societal challenges in terms of the delivery of support and services. Dementia presents one of the most pressing issues in contemporary health and social care. The condition affects nearly 19 million people across Organisation for Economic Development (OECD) countries and accordingly affects millions of family members who often provide informal care: this input from informal carers is estimated to cover from 40% to 75% of total costs, thereby exceeding the cost of formal, professional care [2]. Family carers therefore take on an unacknowledged role in shielding society from further economic impacts of dementia.

It is crucial, however, that dementia is neither reduced to the neurodegenerative basis of the condition nor viewed simply as an economic burden to society. Rather, dementia substantially affects various people involved in different kinds of relationships. Experiences within emerging caring relationships depend on the circumstances and the conditions of the relationship and the persons involved. In that sense, individual experience cannot be entangled from situational and sociocultural influences. This contribution looks at male spousal carers and the conditions that shape their experiences of caring for partners with dementia.

Concepts such as personhood [3] highlight how the effective support of people with dementia must be undergirded by a recognition of the relational and social dimensions of experience. However, this relational and social understanding of the experience of dementia must be attuned to the specific circumstances of those experiencing the condition and its impact. For example, personal characteristics (and the associated sociocultural definitions of these characteristics) will shape the experience of dementia and care. The negative aspects of the experience of being a carer for a person with dementia are often framed as a ‘burden’. However, the experience of this burden is, aside from the particular symptoms of the condition, strongly shaped by social circumstances. An objective burden arising from care needs and challenges linked to the condition is experienced subjectively in different ways [4]. Ku et al. [5], for instance, emphasise the strong impact of socially shaped conditions such as financial strains on the experience of a burden. But carers’ perceptions of the situation, the quality of care and the social impact also contribute to the experience of a burden [6,7]. The concept of ‘social location’ highlights the importance of sensitivity to the complexity of people’s personal (and relational) circumstances [8,9].

A key element of social location is gender: the experience can only be adequately understood if the gendered basis of care relationships is recognised. “Gender, the complex of social relations and practices attached to biological sex, is one of the most important socio-cultural factors influencing health and health-related behaviour” [10]. However, it is an underexplored factor within dementia studies [11,12]. Within academic research, people with dementia are often presented in gender-neutral terms [13]. This limits the scope for the relational and social basis of the experience to be evaluated effectively, hindering the illumination of how gendered norms and cultural expectations affect the basis of caring. McDonnell and Ryan [14], for example, show how gender differences in the caregiver burden can be observed but conclude that there still is a lack of research on the particular needs and experiences of male carers. However, a simplistic and essentialist perspective of social categories should also be avoided [15]: the experience of male carers will be diverse, and it must also be recognised that the coping strategies identified in this paper will not be exclusively associated with masculinity. Nevertheless, while it is a challenge to identify distinctive gender-related elements of an experience, the alternative approach (overlooking the influence of gender) can only offer a distorted perspective.

Associated with this lack of a gender-specific account of dementia and care, the need to grasp the experience of male carers raises distinctive matters for consideration. Drawing on a constructivist conception of gender [16] and specifically masculinity, we investigate the ways in which gender is articulated in particular relationships and recalled in interview settings. Contemporary societies place a high value upon standards of rationalism and economic productivity [17]. In contrast, different domains, such as unpaid care and dependency upon others, are labelled as inferior [18]. These standards mean that people within care relationships, who are likely to require external support, are at risk of feeling undermined by these cultural standards. While these challenges will affect both men and women, these standards arguably present distinctive challenges to men as masculinity is identified with strength, courage and instrumental competence. This is associated with the patriarchal basis of societies, reinforcing constructions of an idealised, hegemonic masculinity aligned with dominance [19,20]. Alternatively, caring is constructed as a feminine quality, associated with maternal and nurturing qualities [21]. While masculinity is aligned with economic contribution and the ‘breadwinning role’, in terms of the domestic sphere, the prevailing gender ideology requires women to take care of men rather than vice versa [22]. With these societal values as a context, caring is likely to be encountered as a gender-nonconforming phenomenon for men [23]. Debates on caring masculinities [24,25] highlight how traditional conceptions of masculinity need to be challenged to embrace men in caring roles. Through actively ‘performing care’, gender assumptions and roles are inevitably reconfigured and renegotiated.

Baker et al. show that due to demographic changes, more older men find themselves in caring roles [26]. In empirical studies, the experience of male carers has received attention within the academic literature. Baker et al. could not identify exactly how masculinity might affect caring experiences in people’s own accounts [26]. In a qualitative study that included five male carers, Rykkje and Tranvåg found that men adapted positively to their circumstances and expanded their repertoire of responsibilities [27]. Men acknowledged the rewards of caregiving, which helped to render their ongoing life situation manageable. Knutsen and Råholm undertook a phenomenological study of male carers [28]. This study captured some of the tensions of caregiving, with men experiencing difficulties relating to grief and loneliness, but also more affirmative aspects of caring associated with love and togetherness. This therefore captures some of the emotional challenges and complexities of caring. In a further qualitative study, husbands caring for their wives who experienced memory loss had to adapt marital roles as they took on caring responsibilities [29]. This meant they had to negotiate new types of relationships. In their grounded theory study, Brown et al. assert that help-seeking behaviours are complex and gender-specific [30]. Choices of action/interaction strategies were linked to the overarching theme of ‘doing the best I can’. This demonstrates a sense of striving to meet perceived societal values, reconciling individual agency with support-seeking initiatives.

In a study that was not dementia-specific but did consider masculinity in relation to the approach to caring adopted by older men, Kluczyńska shows how men’s responses to care responsibilities varied [31]. This was influenced by how men defined their responsibilities in relation to masculinity: men who felt they had slipped down the hierarchy of masculinity felt like failures, whereas those who were able to reconcile their masculinity with their new roles reported more positive experiences. Regarding domestic roles specifically, Boyle found that male carers were often reluctant to take on housework [22]. When these roles were taken on, it was often out of necessity rather than any sense of a requirement for gender equality. In addition, in a study that addressed how male carers negotiated formal support [32], it was found that a fear of perceived failure and a loss of control contributed to their low trust in, and dissatisfaction with, accessed services. Men’s desire to be seen as competent in their caring role and to remain involved in decision-making processes is also asserted. Further to these studies, from a secondary analysis of qualitative interviews, Hellström et al. demonstrate how men adopt and normalise the caregiving role, developing and internalising a lexicon related to their new responsibilities [33]. It is appropriately asserted that gendered assumptions about men and caring should not supersede attention to the individual needs of male carers. However, it can also be argued that this is not an either/or matter: recognising an individual’s needs must be reconciled with recognising the influences of social location that inhere within personal experience.

This paper builds on this literature by addressing the following research question: how do male carers of women living with dementia navigate the impacts of the condition?

## 2. Materials and Methods

To address this question, we adopted a ‘Doing Gender’ approach [16] by inviting participants to discuss their experiences in their everyday settings. We therefore chose a joint interview approach in which both the person with dementia and the spousal carer were present and would contribute to the interview. ‘Doing gender’ within this setting means that experiences and thoughts are expressed in the presence of the partner with whom people live their everyday lives. The gender-oriented basis of this study therefore provides a valuable contribution to an under-researched dimension of the experience of dementia and care. A qualitative research design was employed that drew on the analytical principles and techniques of thematic analysis and was situated within the paradigmatic framework of constructivism and interpretivism.

### 2.1. Recruitment and Sample

This qualitative research set out to recruit mixed-sex couples in which the woman had been diagnosed with dementia. Ten couples were recruited in which a male spouse cared for a woman who had dementia. The study was based in the West Midlands region of England. Couples were recruited from two different dementia support groups (*n* = 9) and via a church organisation (*n* = 1). Invitations and information sheets were shared by key contacts within these organisations with those who met the inclusion criteria. The couples could then contact the researcher to arrange a discussion to explore the nature of participating in the research. If both members of the couple were happy to take part, an interview was arranged. Table 1 below shows the characteristics of the male carers who participated. All the men had retired from work, but their former occupations are highlighted in the table. The ages of the carers are also shown in the table, and these spanned from 62 to 86. All the participants were white and British. While the analytical focus of this paper is gender, it must be recognised how other dimensions of social location, such as age and ethnicity, will also influence the experience of caring.

Some of the characteristics of the partners of the male carers are also shown in the table. There was no exclusion criterion regarding the type of dementia, and the majority had Alzheimer’s. The timescale following diagnosis varied across the participants. It is important to acknowledge that proximity to diagnosis is a factor that will affect experience and shape experiential accounts. The researcher also made a note of the apparent level of understanding demonstrated by each female partner during the interviews and their scope to engage with the process. This subjective judgement of the severity of dementia shapes the classification (mild or moderate) included in the table. The names of all participants have been changed in this article.

### 2.2. Data Collection

Men took part in semi-structured joint interviews alongside their partners. The participants were interviewed twice to seek extensive experiential insights, with a six-month interval between these interviews. While this dual-interview approach was adopted, the aim was to generate depth of insight rather than longitudinal data for comparison. The interviews from these two time-points were therefore combined for the purposes of analysis. Audio recordings of the interviews were obtained, and this audio material was then transcribed verbatim. The interviews took place in the participants’ family homes apart from one interview that took place in a residential care home. For reasons related to health, one couple did not take part in the second interview. A total of 19 interviews were therefore undertaken. The mean duration of the interviews was 67 min.

The interviews followed a flexible schedule in line with the research aim, which allowed respondents to raise matters that were of salience to them. The topics addressed included negotiating the spousal relationship; navigating relationships with members of the broader family, neighbours and friends; and accessing formal services and support. Written informed consent was obtained from all participants prior to the commencement of each interview. The interviewer was cognisant of the requirement for all interviewees to feel comfortable within the interview situation. The interviewees were aware that they could take a break from the process at any point. If any of the interviewees had found the process distressing, then the interview would have been immediately discontinued. The Staffordshire University Research Ethics Committee provided ethical approval for this research.

### 2.3. Data Analysis

A thematic analysis enabled the principal themes from the data collection to be identified, establishing the key content and the pertinence of experiential matters for the participants [34]. The first stage of the analysis required listening to the audio of the interviews and reading the transcripts. While this article focuses on the experiences of male carers, due to the conversational nature of the joint interviews, the male carers’ perspectives were entangled with those of their partners. The next phase of the analysis was therefore to focus on the personal accounts of the male carers within the transcripts. A system of coding was then applied wherein notes and labels were applied to the text. These were then evaluated and organised into themes that convey the key experiential perspectives expressed by the male carers. The following key themes are explored below: making sense of the condition; treating dementia as a problem to be solved; and engaging with professionals and support.

In the findings, an ellipsis in square brackets highlights that some text has been removed from a quote. This is to aid the clarity of delivery and does not alter the meaning of the participants’ statements. A degree of ‘cleaning’ the text is compatible with the rigorous delivery of qualitative data [35].

### 2.4. Limitations

A sample size of 10 was deemed appropriate, considering the complexities of the joint-interview approach. This was the intended sample size, which was achieved via the recruitment process outlined above. When this sample size was achieved, it was considered that no further recruitment was required as sufficient data had been obtained to address the research aim. Joint interviews generate multiple dimensions for analysis, including subjective perspectives and how these are interwoven in conversation. The dual-interview approach, with the participants interviewed on two occasions, also increased the scope and depth of the data. This sample size therefore generated a very substantial, but manageable, dataset. However, it must be recognised that the nature and scale of the sample will shape the results and, accordingly, no claims to generalisability are asserted. The findings should have a degree of transferability [36] as the emotional and practical challenges of informal care will have some commonalities across different settings. However, the basis of the sample and its social and cultural context must be considered: the emphasis of this study is on male carers (who were all white and British).

As noted above, the joint-interview approach allows for access to the perspectives of two participants and how they are co-constructed within conversational exchanges. Disaggregating the views of the male carers from the joint interview enables an in-depth evaluation of the factors shaping their experience of caring. This does not understate the value of the perspectives of the women living with dementia. It is a limitation of this paper that the views of the women living with dementia are not addressed (the perspectives of the women living with dementia have been addressed elsewhere—see [37]). What is being asserted is the value of an undiluted focus on the perspectives of the male carers who participated in this research. What must be recognised is the contextual basis of the data collection in which these views were expressed.

The relational basis of the interview setting (which included the male carer, the woman with dementia and a male interviewer) will have shaped the nature of the respondent accounts [38,39]. While this recognition is vital, obtaining views within a joint interview setting offers distinctive value and can even contribute to the authenticity of the perspectives expressed [40]. There is no such thing as a hermetic ‘individual account’ which is free from contextual influences and pressures. The contextual influences within a joint interview are to some extent commensurate with the social and relational circumstances of the male carers (in a spousal relationship), as many situations they encounter will be negotiated alongside their partner [41].

## 3. Results

The male carers shared their experiences and thoughts on the situation, reflecting on themselves, their partners and their relationships. Their reflections took place in conversation with the interviewer and their partners. Doing gender, i.e., expressing different forms of masculinity, can therefore be observed within the very relationships that shape people’s everyday lives. Below, we present three themes that emerged that exemplify the challenges associated with the articulation of gender identity within care discourses.

### 3.1. Making Sense of the Condition

In the quote below, Keith discusses how he seeks to make sense of his partner’s condition. He uses a comparison with balls in a lottery being jumbled up but then placed in a sequence to capture how he feels his wife’s brain is functioning. This shows an endeavour to understand the disease in spatial and mechanistic terms. The ‘interior’ nature of dementia, in which external physical indicators of illness are often absent, could lead carers to apply such sense-making interpretations. There is also the sense that this is an ongoing activity and that working out the underlying basis of the condition is a necessary task for the carer.

*I explain to people what it is. I think of it as the lotto on the telly where the balls go round and then one drops out and all the numbers are mixed up and then all of a sudden they just flash and it’s ‘one, two, three, four’, whatever. I think that’s what’s happening in there and it’s just trying to work out how.*

(Keith)

As well as lacking external physical markers, dementia also presents a challenge to carers as it is difficult to predict how the condition will progress for any individual. This reduces the potential to manage or control the impacts of the condition. In the quote below, Stuart notes how this uncertainty affects their lives: a lack of clear temporal markers prevents them living beyond a ‘day-to-day’ mode of existence.

*So really it’s just virtually live each day as it, you know, as it comes really; because you don’t know where you’re going to be. There’s no sort of, er, it’s like if you’ve got cancer, they turn around and say, oh, well you’ve got six months to live or something like that, there’s nothing like that and nobody knows how it’s going to progress, so you’ve just got to go with what you’ve got.*

(Stuart)

In the quote below, Liam highlights how he made sense of the condition by discussing it with his partner. There appears, therefore, to be a clear relational basis to the sense-making process. Liam also asserts that extensive life experience prepared them for this situation. While the couple spoke openly about the condition and its impact, for a period, they operated in a closed awareness context [42] with respect to the disclosure of the diagnosis to others, including their children. The dismissal of others’ concerns could align with a masculine sense of independent coping. However, in this instance, it does have a relational dimension as he asserts how their approach to the condition has brought them closer together:

*We’ve both got enough experience in life sort of thing you know to put up with what’s thrown at us and when she got diagnosed we sat and talked about it for months before telling the kids or anything like that you know and we do, we laugh and joke. Okay, people, if they don’t like it, well that’s tough, we have a dance in the kitchen, we do don’t we, and it’s brought us so much closer together you know.*

(Liam)

The diagnosis of the condition can also help men to make sense of their circumstances. Below, Nick highlights that he felt he lacked patience with Emma. Carers may believe that the person with dementia is deliberately trying to antagonise them when their behaviour is a consequence of cognitive impairment [43]. However, the diagnosis of the condition helped Nick to recognise that dementia was affecting Emma’s behaviour. This recognition helped him to be more understanding, and there is also a sense of duty apparent in his statement, ‘that’s what you have to do’.

*I didn’t always have patience as Emma said. When we, when we didn’t know what was causing our arguments over stupid things, I didn’t realise part of it was, was the Alzheimer’s, but having found out that that’s what it was and to be more understanding, I mean, that’s what you have to do.*

(Nick)

The impact of dementia also calls attention to the wider matters of which men need to make sense. The men highlighted how this led to a change in domestic roles, meaning that they often had to address household tasks that had been hitherto unfamiliar. Giles’s quote below captures this point. Giles refers to his lack of experience but also newfound abilities. It is also notable that he refers to ‘professional’, thereby aligning his capability with the terminology of paid employment. This therefore invokes a traditional male perspective on work in relation to domestic duties. The reorientation of tasks with ‘male’ values could help to mitigate against any frustrations encountered with these new roles [31].

*The other area which I’m now a number one professional, if you like, which is, I wouldn’t have known where to start, six months ago, I do err the washing, put the washing machine on, dryer on, etc.*

(Giles)

The challenges of making sense of the condition and its impacts can also have emotional effects upon male carers. As Norman conveyed the point below, he became openly emotional. His wife’s dementia makes him feel incompetent. He is also unsure how to seek help. Competence is a gendered trait [23], and men might encounter particular emotional challenges when they feel unable to cope with practical difficulties in an independent manner.

*So you know, I just feel incompetent to deal with it, because I just don’t know what to do. I mean there’s plenty of people offering help, but when it comes to taking it, where do you go?*

(Norman)

### 3.2. Treating Dementia as a Problem to Be Solved

The way that men make sense of the condition relates to another dimension of their care experience. As dementia can be approached by male carers as a technical matter, it can also be addressed as a problem to be solved. In the quote below, Geoff discusses a potential treatment he has accessed that is not available via prescription. There is a sense of independent research and endeavour to Geoff’s approach, as well as conveying the investment of resources required and the negotiation of a better financial deal:

*Souvenaid, it’s a one a day drink, and apparently it had been medically tested and so many people that have tried it claimed that the, it may have slowed the process down a little bit, somewhat. Some reported different reactions on their speech and their memory, and whatever. So I thought, “Right, we’ll give it a go.” […] I found the company and then I got it on the internet. And I ordered it through the internet because you couldn’t, it was not on prescription. And it was £200 and, there was £50 off if you got three boxes.*

(Geoff)

An approach adopted by Giles provides a further example of a man seeking measures to address the direct impacts of the dementia. Giles has obtained a book that offers potential courses of action that can mitigate the influence of dementia. It appears that Giles’s son is sceptical of the credibility of this text, but Giles refers to a cost–benefit calculation he has undertaken which means he will pursue some of the recommendations offered by the author:

*I’ve got this thing called, “The End of Alzheimer’s”, book, which my son, in conversation says, “It’s not proven”, but as I said to him “Well if we do some of the things that are in that book, and it doesn’t do us any harm, err what have we lost?”*

(Giles)

Further to the quote above, Giles queries the input being offered by health professionals in terms of medication. He feels that wider possibilities in terms of interventions could be offered by professionals, even if their efficacy is uncertain. The book he has obtained offers such possibilities. Dementia presents a challenge to carers seeking to support their partners as there is no cure, and treatments for types of dementia such as Alzheimer’s are limited to addressing symptoms only. This might present challenges for male carers who might prefer instrumental methods for tackling a situation and with dementia, feel that their agential scope to ‘fight back’ against the condition is highly circumscribed.

*What I don’t understand is why they don’t seem to want to help other than a basic tablet […] but why they don’t tell you, are these possibilities? Even if they aren’t, they don’t work, but I think you should be told, and a lot of things in, in this book are about checking your balances of certain vitamins and things which may affect, may affect Alzheimer’s.*

(Giles)

The requirement for active intervention is also indicated by Keith. For example, if his wife could not remember a name, he would work through a routine with her to try and prompt her memory (this tactic was apparent during the process of the joint interview.) Rather than providing an answer directly to his wife, this endeavour set out to ensure she was using her brain to enable recall, with an overarching aim to counter the impacts of the condition. This strategy to boost cognitive performance demonstrates a problem-focused coping approach to the care scenario (see [44]). Keith feels that his striving, and the encouragement of his partner, will help her to improve. He also makes a comparison with other male carers and deems that they are too acquiescent:

*We’ve got friends and their husbands can’t cope with them and they say, “Yes sir, no sir, three bags full sir”. Now I won’t let that happen, I’m a bugger, I just won’t let it. I want her to get better you see. I keep pushing and pushing.*

(Keith)

In the following quote, Keith further explains the challenges they have encountered and states how his partner was initially reluctant to listen to his advice on how to tackle the condition. However, he feels the situation has now improved as she wishes to get better. It is hoped that an active approach to tackling the condition can help them to get their ‘lives back’:

*She always has been stubborn and I think two things, at first she was stubborn because it was frustration and she didn’t know what to do and how to go about it and she wouldn’t listen, she wouldn’t learn and everything else. All that has all kind of gone and now because she wants to get better so it’s a go-go, I think anyway because you do don’t you? […] We want to get our lives back don’t we?*

(Keith)

It is important to recognise that the approaches of the men to the condition were diverse. While strategies to ameliorate the impacts of dementia, or even cure the condition, were apparent in the accounts, other orientations to caring were also identifiable. In the quote below, Nick refers to the requirement for understanding and patience. However, he also acknowledges and accepts limits to the scope for his understanding as he does not have the condition:

*It’s two words, it’s understanding and patience. It’s very hard, I, I would be honest with you it’s very hard to have the patience, but that’s what it is, that’s what caring is, it’s having patience to try and look after people, trying to understand which I will never understand because I ain’t got it, you know, you know, it’s impossible for me to understand, just to do your best to understand.*

(Nick)

### 3.3. Engaging with Professionals and Support

While the previous themes address the personal endeavours of the men to make sense of and ‘solve’ the problems associated with the condition, this final theme explores how the men engage with external support. In response to a question about whether he is satisfied with the level of support offered, Mark highlights that he feels uninformed about the inputs that have been provided by health professionals. It appears a tablet was prescribed and then stopped, and Mark was unsure as to both the nature of this medication and the reason it was discontinued.

*Not really, because he didn’t tell us anything about it you know, “Take these tablets”, he didn’t say what they were for, and then they stopped them, and they didn’t tell us why they stopped them. I mean I don’t know what tests you can do to test for dementia.*

(Mark)

Similarly, Geoff highlights the limitation of interventions. He discusses the questions presented to his wife, Fiona, during a cognitive function assessment. An emphasis on medication and cognitive function assessments from some male carers shows how technical and practical orientations therefore remain prominent when men engage with professional support. Geoff notes that this is repeated on different visits and queries the value and validity of this assessment format (see [45]). Geoff highlights that Fiona is able to perform well during the tests, but this does not capture some of the challenges presented by her behaviour during their day-to-day interactions.

*I think one of the problems when they come out, it’s, the, the questions are the same as what they’ve asked before, which Fiona already knows the, the differences that they, they go back on the dates and everything, and backwards and that. […] they’ll say, I mean the, the one girl turned around and said “You’re doing remarkedly well Fiona”, but they don’t see the other side.*

(Geoff)

The value of external input is queried to an even clearer extent by Norman. He discusses support groups and the speakers who are recruited to address these groups. Norman queries the basis of their expertise and contrasts his own direct experience with those who are invited to offer their insights, who seem to lack experience. Norman’s emotional difficulties with feeling incompetent were apparent under the previous theme and he stated he did not know who to approach for support. Despite this challenge, even when support is available, the credibility of the person helping is also important.

*Oh, don’t talk to me about support groups. I’ve been to two or three of them and I find there’s people telling me what to do that have never had to do it themselves.*

(Norman)

A more positive view of support groups is offered by Nick: he asserts the value of a group in which he is involved. He highlights the value of just talking and how this can help when carers might be experiencing loneliness (see [46]).

*Being a carer, I’m sure you’ve been told it before, it’s very lonely existence. People, just the two of them together, who haven’t got an outside family or friends, good friends. So all we do is talk. We talk for two hours and laugh and joke and there’s not many come, about eight people come.*

(Nick)

A further affirmative perspective on support groups is provided below. Keith invests the access of external support with a sense of personal agency and endeavour. His quote highlights that the initial requirement is to want advice, which can then be obtained. This then provides a resource which can help in managing the condition and situation.

*If you want advice, they are willing to give you advice and that’s what I like. It’s not swept under the carpet. We need to know more about it; we need to know how to cope.*

(Keith)

## 4. Discussion

The results indicate some of the key concerns of male carers and the challenges they encounter; the influence of gendered experience and cultural constructions is apparent in the accounts expressed by the men above. By implicitly negotiating conflicting ideals of hegemonic masculinity, the male carers in this study try to link their individual experiences to broader social positionings. As noted in the introduction, traditional cultural notions of masculinity emphasise that autonomy, competence and control shape the experience of men [19,23]. The sense-making process demonstrated in the first theme shows how men will adopt a lens for understanding dementia and its impacts that aligns with these values. This includes conceptualising the disease in physical or technical terms and struggling with a reduced sense of control due to a lack of clear temporality as to the status passage [42] of the condition. The process of making sense of the condition was also shown to relate to autonomous coping in relation to the disclosure of the condition. In addition, if male carers feel unable to measure up to the values of competence and control, then this could place them in an emotionally vulnerable position.

It was also noted that caring means that men experience a change in domestic roles; the extent of this is likely to exceed that experienced by female carers in a similar position. While generalisations should be avoided, older men are less likely to be familiar with domestic roles, such as cooking and cleaning. Male carers are therefore likely to experience additional levels of biographic disruption [47] when their partner is diagnosed with dementia. This can disturb not only practical routines but perceptions of one’s own self-identity. There is likely to be a temporal dimension to this finding. The perspectives of the men above might reflect their position within a cohort. All the men in the sample would have spent the majority of their ‘working age’ within a context in which the man was positioned as the ‘breadwinner’ within the household while women were responsible for childrearing and domestic responsibilities. This distinction has been eroded, with women now engaged much more extensively within the workforce in the UK over recent decades [48]. However, the way this has changed gender roles should not be overstated, and to some extent, a division of roles and cultural standards endures [22].

Conceptualising the condition in physical or technical terms also creates the possibility for male carers to seek to improve or mend the condition. This shifts the nature of responsibilities away from the domain of care and aligns more with the technical and problem-solving sphere of paid employment. This can prompt a rather instrumental approach to addressing their partner’s dementia. It is highlighted that caregivers (regardless of gender) will want to fix the person and make them “normal again” [49] (p. 5). Within care relationships, it is possible that men might be more prone than women to adopting active measures to address this goal. In this research study, direct measures to thwart the progression of dementia (and even cure the illness) were apparent and sought by different means; for example, via dietary measures or memory training. The pursuit of instrumental solutions also shifts the emphasis to cure rather than care. It has been argued that “the traits associated with masculinity do not leave much, if any, room for nurturing tendencies” [23] (p. 197). This, again, reflects a traditional gendered imaginary regarding social roles, with ‘cure’ aligned with medicine and ‘care’ predominantly aligned with nursing: “the nursing profession is viewed as female with low skills, social status, salary, academic level and entry requirements, and with little autonomy” [50] (p. 1).

The challenge of adopting such an approach is that it might be counterproductive. For example, engaging in memory-oriented tasks to prevent cognitive decline has the potential to frustrate the person with dementia and cause them stress. When measures to prevent decline are unsuccessful, it could also present emotional challenges to carers seeking to ‘thwart’ the condition, with this outcome threatening self-perceptions of efficacy and competence. This could, in turn, diminish the quality of the care relationship and have negative impacts for the person with dementia. The endeavour to cure or mend the condition arguably aligns with a ‘defectological’ model that reduces dementia to pathology and dysfunction [43]. This ‘standard paradigm’ strongly influences cultural values that, in turn, shape public perceptions of dementia [51]. In association with this perspective, it has been highlighted that the assumptions of decline and loss associated with dementia can influence carer behaviours. This can even lead to a ‘malignant positioning’ [52], which provides a lens through which healthy others make sense of the behaviour of a person with dementia [53]. This emphasises negative attributes in potentially malignant ways which can undermine the personhood of the person with dementia [3].

The endeavour to improve or mend the condition provides some matters for scrutiny regarding these concepts: a carer who is seeking to ‘fight back’ against the condition is to some extent resisting ‘malignant positioning’. This strategy does not assume a straightforward trajectory of loss or decline and demonstrates a hope, or even belief, that the impacts of the condition can be resisted. Nevertheless, it could be asserted that the locus of attention is still decline and loss, even if the endeavour is to counter these effects. As noted above, this strategy could generate distinctive difficulties for the person with dementia. It is not only assumptions of decline that could contribute to problematic positioning but also a more optimistic view that the condition can be overcome or fixed. It is therefore vital that carer perspectives on the condition are credible. This means recognising the person’s potential to live a good life with dementia, while also recognising the palpable (and irreversible) neurodegenerative basis of the condition. This does place carers in a precarious position as they grapple with the duality of the person and the condition [49]. Being aware of the nature of the condition can help carers to make sense of their partner’s behaviours. However, placing too much emphasis on the neurological basis of dementia could lead to the person being undermined or dehumanised [3].

It is vital to avoid being judgemental of carers who are presented with very substantial practical and emotional challenges. It can also be argued that the terminology of ‘malignancy’ is at risk of contributing to a ‘blame’ discourse in which carers are held responsible for the challenges encountered by people with dementia [54]. What is being asserted is that carers require appropriate support so they are able to make sense of the condition and provide optimal support themselves for their partners or family members. Among other things, this support should recognise the gendered basis of care relationships. It should also be noted that within this sample, approaches to the condition varied. Not all men conceptualised the condition in technical (or fixable) ways. For example, Nick refers to an intrinsic deficit in his grasp of his partner’s situation, as he does not have the condition, and asserts the value of patience and understanding. This shows that while clearly not all men react to the challenges of dementia in a unified way, particular patterns can be dominant. The fact that these patterns are linked to broader gendered, societal assumptions means that the inclusion of a gender perspective is crucial. While other status characteristics, such as class, ethnicity, and educational background. undoubtedly impact the experience of and the reaction to a spouse having the condition, this relation to dominant societal patterns within care relationships highlights the significance of gender as an intersecting factor.

Perspectives on external support also demonstrate how men negotiate the experience of their partner’s condition. Research tends to show that men are less likely to seek support themselves, as seeking help is not readily aligned with a self-sufficient masculine identity [55]. The findings in this paper highlight that when men are carers, engagement with services is complex and diverse. While the responses varied, a masculine-gendered approach can be discerned within this theme. This included being sceptical of the basis of others’ expertise and asserting greater personal experience. It has been highlighted that the hegemonic masculine discourses associated with self-sufficiency can complicate support-seeking endeavours [56]. However, the findings above also indicate how support seeking could be reconciled with such standards of masculinity: support is a resource that can be actively drawn upon to shore up a sense of control and reinforce the perceived ability to resist the impacts of the condition.

While caring and nurturing might be gender-nonconforming for older men, this does not imply that men are inferior carers to women [23]. What is being highlighted is that men are likely to encounter particular (gendered) challenges associated with the caring role. The relational basis of personhood means that the experiences of male carers will have an impact upon the partners or family members for whom they are caring. The findings in this paper highlight that policy and practice must be configured to address the gendered basis of caring. This can help to address the particular challenges that men might encounter. This can include educational initiatives that can promote the types of support that are likely to be most beneficial to the person with dementia. This needs to be based on a credible perspective that asserts a recognition of the neurodegenerative basis of dementia, with the acknowledgement that experiencing the condition is not defined by decline and loss [52].

## 5. Conclusions

This paper conveys the findings of a qualitative research study that addressed how male carers for women living with dementia negotiate the impacts of dementia. The key themes conveyed in the findings assert that masculine-gendered cultural standards influence the perspectives of male carers. Insights from qualitative research designs can always only be partial. The selection of participants, which was based on self-selection mechanisms via support groups, does not allow statements to be made for all male carers. However, the aim of this study lay foremost in the identification of patterns that signify particular experiences, approaches or behaviours linked to conceptions of masculinity and care. While the complexity of the accounts must be acknowledged, the importance of autonomy, competence and control are detectable, and these affect the experience of caring. This shapes sense-making in relation to the condition, coping strategies to address the impacts of dementia and engagement with external support. To ensure the experience of dementia and care is conveyed credibly, sustained research into the gendered basis of care relationships is required. As a chosen methodology, joint interviews proved to be highly useful for this endeavour. The men talked about their experiences and perspectives within the very setting that provides a key orienting context for their everyday lives. Emotional relating and wellbeing largely happen within the men’s spousal relationships. Understanding the couple as a unit that must deal with the effects of dementia [57] asks for methodological approaches that centre on this relational basis. The male carers’ accounts recorded within these settings represent everyday experiences, struggles and moral challenges.

This gender-sensitive approach can help inform policy and practice endeavours to support people with dementia and their carers. Since associations with care and caring practices are highly gendered, support and intervention programmes can fall into the same gendered logics. This does not mean that support offers can be tailored specifically to address all male carers, but sensitivity to different experiences, different understandings of dementia and different approaches to care is crucial in order to tackle the needs of all carers. Policies and practical support can only be partial and inchoate if the influence of gender on the experience of caring is understated or overlooked.

## Figures and Tables

**Table 1:** Participant characteristics.

Name	Age	Occupation	Length of Relationship (Years)	Partner’s Condition	Severity of Partner’s Condition	Time since Diagnosis
Stuart	73	Haulage	43	Alzheimer’s	Mild	2 years
Norman	80	Teacher	58	Vascular	Moderate	1 year
Geoff	69	Skilled manual	47	Frontotemporal	Mild	2 years
Steve	62	Skilled manual	40	Alzheimer’s	Mild	4 years
Nick	72	Sales	48	Alzheimer’s	Mild	9 years
Mark	86	Skilled manual	5	Alzheimer’s	Mild	7 months
Keith	74	Haulage	54	Alzheimer’s	Mild	1 month
Liam	66	Skilled manual	23	Alzheimer’s	Mild	3 years
Giles	71	Accountant	36	Alzheimer’s	Moderate	3 months
Roger	67	Security	20	Alzheimer’s	Moderate	4 years

## Data Availability

For ethical reasons, the full dataset is not being made publicly available.
